# Colorectal Cancer Presenting as Sacral Pain at a Chiropractic Clinic

**DOI:** 10.7759/cureus.39277

**Published:** 2023-05-20

**Authors:** Gabriel Siu Nam Ng, Isabel Si Wing Chow

**Affiliations:** 1 Chiropractic and Physiotherapy Centre, New York Medical Group, Hong Kong, CHN

**Keywords:** quality of life, palliative care, rehabilitation, chiropractic, back pain, colon cancer, spinal metastasis

## Abstract

This case report describes a diagnosis of spinal pain secondary to metastatic colon cancer, highlighting the need for close monitoring and an interdisciplinary approach for this cohort. An 82-year-old female presented with acute exacerbation of chronic low back pain with a rating of 7/10. She had a history of stage III colon cancer diagnosed 17 years previously and was currently in remission. Red flags included worsening pain after four days of oral analgesics and a history of cancer. Imaging revealed an osteolytic L5 vertebral lesion with endplate disruption, consistent with metastasis. She was urgently referred to an oncologist who ordered chemotherapy and radiotherapy. The metastatic spread of malignancy to the spine can manifest as new or progressive back pain and requires prompt diagnosis and management. Magnetic resonance imaging is recommended for the detection of osseous lesions and spinal cord compression. The case report serves as an educational tool for chiropractors in recognizing and managing spinal metastasis. By sharing this case report, healthcare professionals can learn from the experiences and challenges faced during the patient's care and apply that knowledge to their practice.

## Introduction

Low back pain (LBP) is the most common condition that chiropractors treat as primary healthcare practitioners [1]. Chiropractic is a distinct healthcare profession focusing on the structural and functional changes of the spine and nervous system [2], and chiropractors are well educated to diagnose and treat neuromusculoskeletal disorders [3]. Although patients with life-threatening LBP rarely present to chiropractic clinics [4], many serious cases have been reported in Hong Kong involving patients seeking chiropractic care when they were unaware of the grave nature of their musculoskeletal symptoms [5-14]. Although the most frequent location for bone metastases from colorectal cancer is the spine [15], it is challenging to diagnose metastases based on clinical presentation alone, and chiropractors must maintain a high index of suspicion for "red flags" [16], such as pain disproportionate to clinical findings, pain at rest, and unintentional weight loss.

Prompt and accurate diagnosis of spinal metastasis relies upon radiological assessment, with magnetic resonance imaging (MRI) proving to be superior to computed tomography (CT) or radiography for the detection of osseous lesions and cord involvement [17]. Referral to a cancer specialist is recommended when diagnostic imaging is beyond the scope of standard chiropractic care or where a metastatic process is confirmed [18]. Before initiating treatment for spinal metastases, it is crucial to rule out primary tumors/cancers that would necessitate an entirely different therapeutic approach [19]. The therapy and management of spinal metastasis necessitate a multidisciplinary approach, including radiation therapy, radiopharmaceuticals, high-intensity focused ultrasound, electrochemotherapy, vertebroplasty, acetabuloplasty, minimally invasive surgery, embolization, and thermal ablation techniques [19]. Quality of life is a priority for patients with metastatic disease [20]; thus, an interdisciplinary approach incorporating medical and rehabilitation strategies may prove most effective for pain reduction and optimization of patient function.

This report describes a rare case of an elderly female with a history of colon cancer who presented to a chiropractic clinic in Hong Kong, with leg weakness and severe sacral pelvic pain and was diagnosed with metastatic disease of the lumbar spine via advanced imaging. It is crucial for chiropractors to quickly identify patients with metastases and direct them to the appropriate follow-up therapy.

## Case presentation

An 88-year-old female presented to the chiropractic clinic in Hong Kong, with a 10-day history of lumbosacral pain radiating to the left lower extremity and accompanying paresis, rated 7 on the 0-10 numeric pain scale. She reported a medical history of cerebrovascular disease, essential hypertension, diabetes mellitus type 2, status post-laparoscopic sigmoidectomy for colon carcinoma 17 years ago, and total abdominal hysterectomy with bilateral salpingo-oophorectomy. Current medications included warfarin, metformin, atorvastatin, omeprazole, and aspirin. She denied experiencing headaches, dizziness, or changes in bowel or bladder functions.

**Figure 1:**
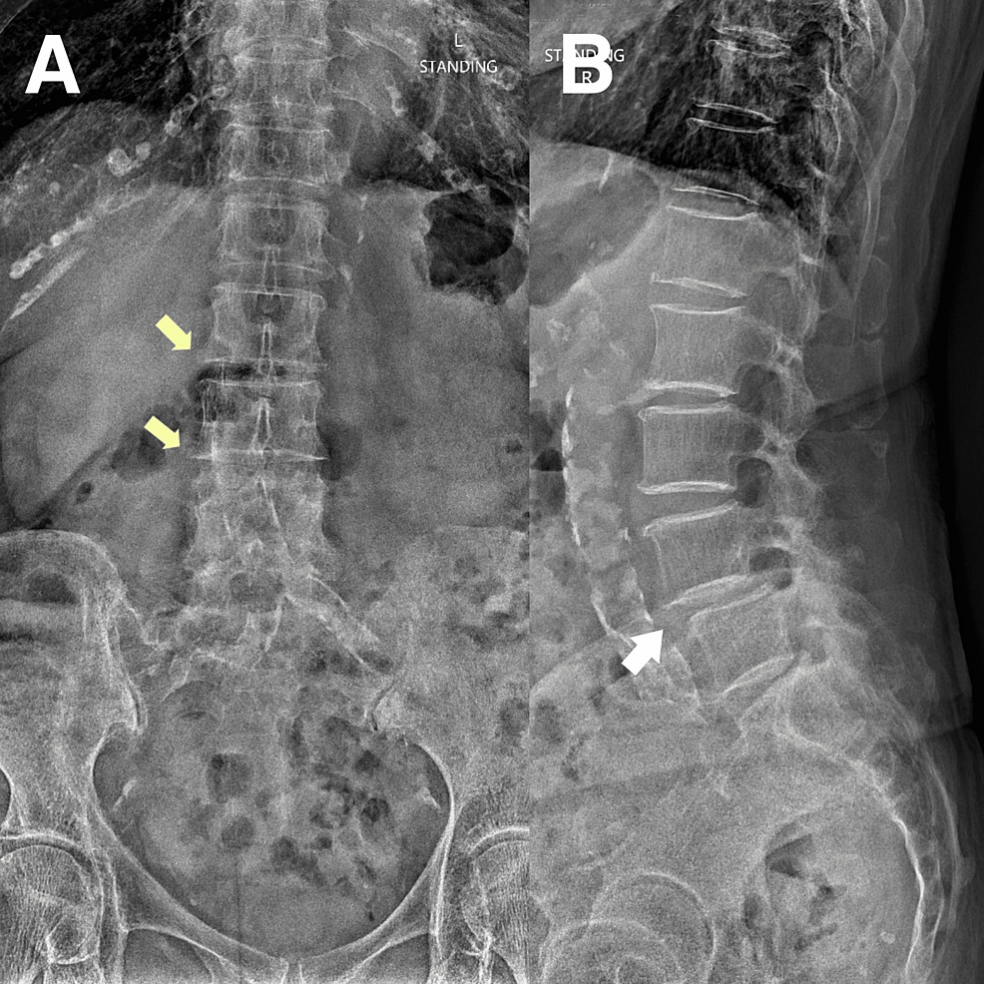
Lumbar radiography A) Anteroposterior view: Marginal osteophytes noted along the lumbar spine suggestive of spondylosis. B) Lateral view: Grade I spondylolisthesis noted at L4/5 and L5/S1. Decreased disc spaces noted from L4-S1 levels suggestive of disc degeneration.

One month before the presentation, the patient presented to the emergency room with acute left leg paresis. Orthopedic consultations and radiographs of the lumbar spine confirmed the diagnosis of spondylolisthesis and degenerative joint disease (Figure 1). She was admitted for two days of intravenous corticosteroid therapy, which provided partial symptomatic relief. Upon discharge, the patient was prescribed oxycodone (2.5 mg), acetaminophen (325 mg), and gabapentin (100mg) for the residual radicular pain. The patient attempted a four-week course of physiotherapy, including stretching and strengthening exercises, without any sustained benefit. She then sought chiropractic care for her LBP.

Upon presentation to the chiropractic clinic, the physical evaluation revealed a limited range of motion in the lumbar extension and left lateral flexion that exacerbated the lumbar and sacral pain. Palpation revealed tenderness in the left sacroiliac joint. Neurological examination demonstrated a positive straight leg raise test and a Bonnet test at 45° on the left side. Motor strength in the left L1 to L3 distribution was rated at 4 out of 5 on a numerical scale due to pain. Based on the clinical presentation of left-sided sacral pain, left leg weakness, and neurological findings, the main differential diagnoses were lumbar radiculopathy, lumbar spondylolisthesis, sciatica, a spinal tumor or metastasis, and hip or sacroiliac joint dysfunction. The chiropractor suspected metastasis, especially because of the patient history of cancer, and ordered MRI to probe the structure of the lumbar spine.

**Figure 2:**
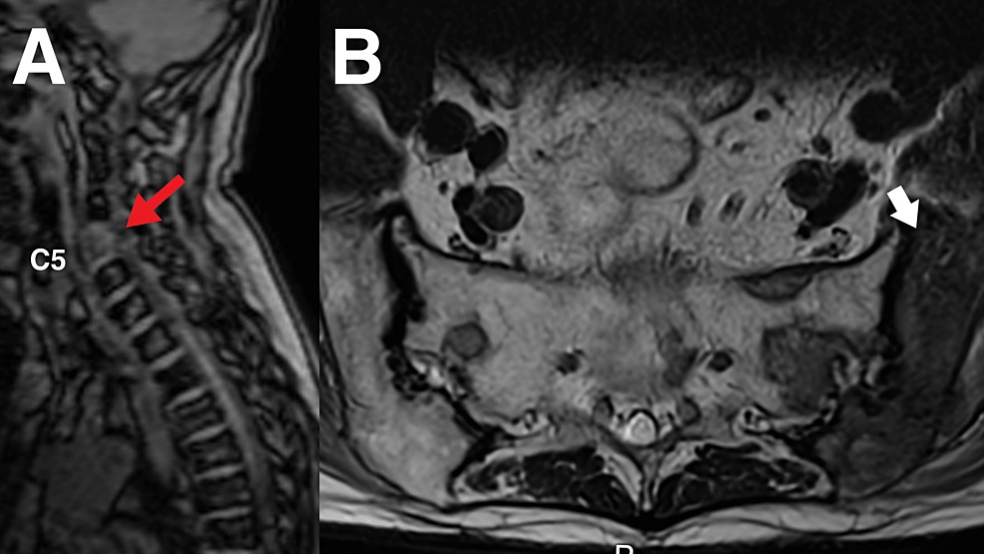
Magnetic resonance imaging (MRI) of the lumbar spine A) An equivocal lesion is seen at C5 vertebral body (red arrow) on initial localizer radiographs. B) several ill-defined bone lesions are seen on the left of the ilium (white arrow) in this MRI that indicate a diagnosis of strongly suspected bone metastases.

**Figure 3:**
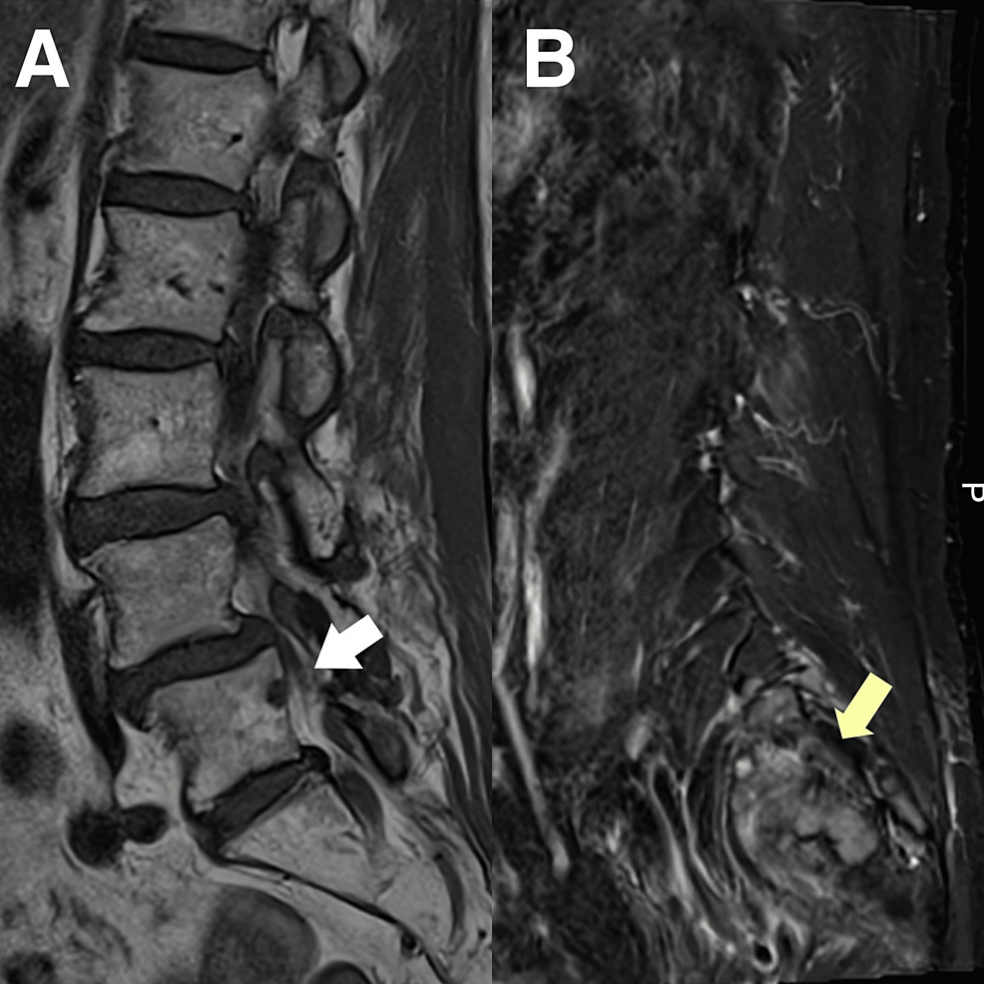
Magnetic resonance imaging (MRI) of the lumbar and sacral spine A) T1W hypointense bone lesions are seen at  L5 vertebral body (white arrow). Grade l L4/5 anterolisthesis. Mild-to-moderate lumbar spondylosis with L2/3, L4/5, and L5/S1 disc height loss. B) Several ill-defined lesions are located at the sacral region (yellow arrow). Collectively, these features are indicative of a diagnosis of strongly suspected bone metastases.

MRI identified an equivocal lesion in the C5 vertebral body and suspicious osseous lesions in the left ilium (Figure 2), L5 vertebra, and sacrum indicating metastatic disease (Figure 3). Additional findings included L4-5 spondylolisthesis, multilevel spondylosis, disc degeneration with annular fissures, neural foraminal stenosis, and thecal sac impingement that could have contributed to the patient's symptoms. The patient was immediately referred to the Medical Oncology Department for further evaluation.

**Figure 4:**
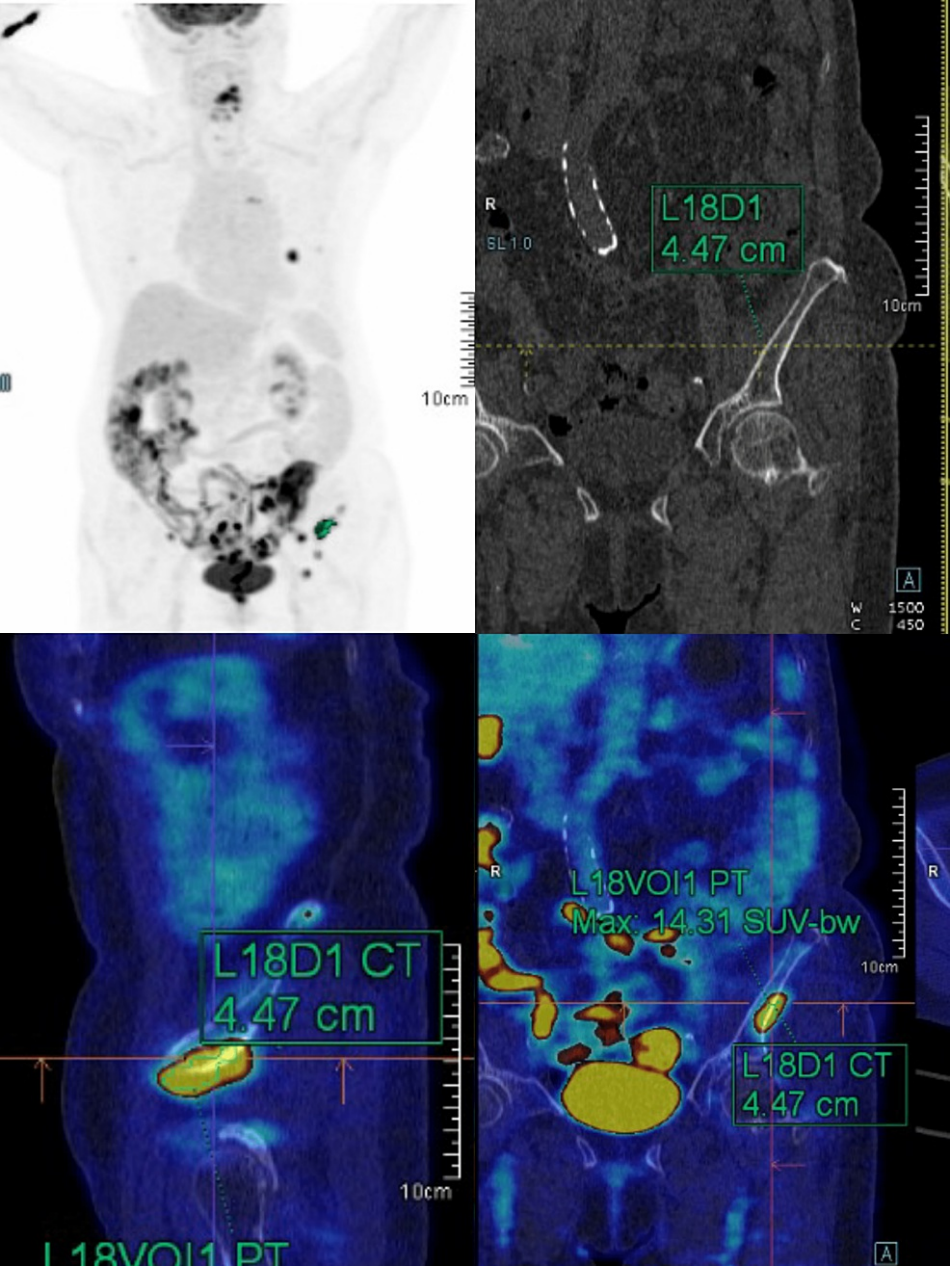
Whole-body PET-CT Sixty minutes after the intravenous injection of 18F-FDG, a non-contrast CT was obtained from the base of the skull to the upper thigh for attenuation correction and anatomic localization of radiotracer activity. Emission PET and post-contrast CT scans were obtained from the same anatomical regions. Images were reconstructed and used to identify a hypermetabolic lung nodule (1.91 x 1.6 cm/SUVmax 15.61), shotty aortopulmonary lymph nodes (0.57 cm/SUVmax 4.55 and 0.67 cm/SUVmax 6.17), and hypermetabolic skeletal lesions on the left ilium: (4.47 cm/SUVmax 14.31). These findings suggested lung, nodal, and bone metastases. PET-CT, positron emission tomography-computed tomography; FDG, fluorodeoxyglucose.

Positron emission tomography-computed tomography (PET-CT) revealed a hypermetabolic lung nodule (1.91 x 1.6 cm/SUVmax 15.61), shotty aortopulmonary lymph nodes (0.57 cm/SUVmax 4.55 and 0.67 cm/SUVmax 6.17), and hypermetabolic skeletal lesions on the left ilium (4.47 cm/SUVmax 14.31) (Figure 4). CT-guided biopsy of the sacral lesion confirmed the diagnosis of adenocarcinoma. Upon further examination, the patient reported a 15-pound unintentional weight loss over three months and recurrent abdominal pain with alternating diarrhea and constipation. Colonoscopy revealed a malignant obstructive mass in the descending colon.

Treatment options include palliative systemic chemotherapy to reduce tumor burden and prolong survival and palliative radiotherapy for bone metastases to reduce pain and the risk of pathologic fracture. Given the patient's symptoms, radiotherapy was initiated for sacral and left iliac metastases. Chemotherapy (folinic acid, 5-fluorouracil, and oxaliplatin) was then begun on the patient and, after six cycles, restaging scans showed a partial response to metastases; however, residual bone pain prompted additional radiotherapy to L5, and this provided significant analgesia. The patient continues close contact and regular follow-up with the medical oncology department and repeats imaging to monitor treatment response and detect disease progression. However, specifics of the colon examination and related findings were not available to the authors, given the limitations of the chiropractic scope of practice.

## Discussion

This report describes an elderly female patient in relatively good health who presented with acute exacerbation of a chronic lumbosacral malady to a chiropractic clinic. The condition of the patient was ultimately diagnosed as a recurrent adenocarcinoma of the colon with osseous metastases upon radiological evaluation and confirmation via CT-guided biopsy. Given the patient history of intermittent mechanical low back discomfort, metastatic infiltration of the fifth lumbar vertebra possibly occurred several months prior to the current presentation but remained occult on prior radiographic imaging. However, radiography lacks sensitivity in the detection of neoplastic lesions, with a minimum bone mineral density loss of 30-50% for radiographic visualization [21].

Upon examination, the patient exhibited various warning signs suggestive of serious pathology in addition to her oncologic history, including qualitative changes in symptomatology with pain refractory to analgesic therapy. This warranted further testing via MRI that revealed the underlying etiology of her lumbar symptomatology. The lumbosacral maladies of this patient were most likely secondary to osteolytic destruction and remodeling changes within the L5 vertebral body, consequent to colon cancer metastasis. Despite the presence of two small lumbar disc protrusions, the patient lacked neural foraminal or central canal compromise to substantiate the radicular pain. Sclerotomal discomfort from the L5 metastatic deposit possibly accounted for her clinical presentation. However, as the patient was not fully managed and treated by the chiropractor, identification and monitoring of metastatic lesions on imaging would be beyond the scope of a chiropractor's training and role.

Following a search of recent review papers, the Index to Chiropractic Literature, Scopus, PubMed, and Google Scholar, many cases of spinal metastases were found involving diagnosis via chiropractic clinics [22-25]. However, we could not identify any case reporting patients presenting to a chiropractor with undiagnosed colorectal cancer. The clinical presentation in this report provided many clues for the chiropractor to suspect metastases, including the presence of spinal pain that was unresponsive to analgesics or physiotherapy, and a history of colon cancer. When patients with a history of malignancy present with new-onset mechanical LBP, an MRI of the lumbar spine is recommended over CT or radiography for optimal visualization of osseous and soft tissue structures [16]. MRI with and without intravenous contrast is a superior diagnostic tool for elderly patients with LBP [26]. When the scope of chiropractic practice precludes this, a prompt referral to an oncologist is recommended for patients with a suspected neoplastic condition.

## Conclusions

In conclusion, this case illustrates the importance of a high level of suspicion for serious pathology in patients with a history of cancer who present with new or worsening spinal pain. Prompt radiological evaluation with MRI is recommended for the optimal visualization of osseous and soft tissue structures. Patients with spinal metastases may present at the chiropractic clinic and require close follow-up and monitoring to assess the therapeutic response and disease progression. A multidisciplinary approach involving the medical and radiation oncologists assigned to the patient may prove most efficacious for the management of pain and optimization of the quality of life in these patients. Future research should aim to establish evidence-based guidelines for the diagnosis and management of spinal metastases, including the evaluation of targeted rehabilitation strategies tailored to patients with comorbid oncologic disease.
